# Case Report: Response With Immunotherapy in a Patient With Mixed Neuroendocrine Non-Neuroendocrine Neoplasms of the Gallbladder

**DOI:** 10.3389/fonc.2021.770156

**Published:** 2021-11-18

**Authors:** Chao Liu, Xiangwei Hua, Zhen Yang, Yuan Guo, Liqun Wu, Jinzhen Cai, Ling Li, Yaxuan Zhang, Ning Fan

**Affiliations:** ^1^ Liver Disease Center, The Affiliated Hospital of Qingdao University, Qingdao, China; ^2^ Department of Pathology, The Affiliated Hospital of Qingdao University, Qingdao, China; ^3^ Medical Department, Yinfeng Gene Technology Co. Ltd., Jinan, China

**Keywords:** case report, MiNENs, a PD-1 inhibitor, sintilimab, gallbladder

## Abstract

**Background:**

Primary neuroendocrine tumors of the gallbladder (GB-NETs) are rare, accounting for 2% of all gallbladder cancers. Among GB-NETs, mixed neuroendocrine non-neuroendocrine neoplasms of the gallbladder (GB-MiNENs) are sporadic.

**Case Presentation:**

A 56-year-old woman admitted to our hospital due to right upper abdominal pain of 3 days duration. She underwent positron emission tomography/computed tomography, which showed multiple metastatic tumors and was unsuitable for operation. Initially, the patient was diagnosed with gallbladder adenocarcinoma. She underwent PD-1 inhibitor or combined with chemotherapy considering the PD-L1 high positive score. In the latter, the patient has the opportunity of surgery, and the new diagnosis was MiNENs. She achieved long-term disease control and has been alive from the first diagnosis.

**Conclusion:**

This case might support the strategy that PD-1 inhibitor could provide a feasible treatment option for MiNENs of gallbladder patients with the positive expression of PD-L1 in the future.

## Introduction

According to 2019 WHO Classification of Tumors of the Digestive System, mixed adenoneuroendocrine carcinoma renamed mixed neuroendocrine non-neuroendocrine neoplasms (MiNENs). MiNENs is mixed epithelial tumors containing both neuroendocrine and nonneuroendocrine components, and they can be distinguished by histological morphology and immunohistochemistry. Furthermore, each component accounts for at least 30% of the tumor burden (1). The study found MiNENs of the gallbladder were rare and had a poor prognosis (2). However, mixed neuroendocrine–nonneuroendocrine neoplasms of the gallbladder (GB-MiNENs) are sporadic. At present, specific treatment guidelines have not been established because of the rarity. There are two main management methods. The first is the latest WHO recommendation to treat MiNEN as adenocarcinoma, and the second is to treat this tumor based on more aggressive histological components (3). Therefore, it is urgent to develop an effective therapy for gallbladder MiNENs. In this report, we describe the benefits of sintilimab combined with chemotherapy in patients with gallbladder MiNENs who have metastasized to the liver after complete surgical resection.

## Case Presentation

**Figure 1:**
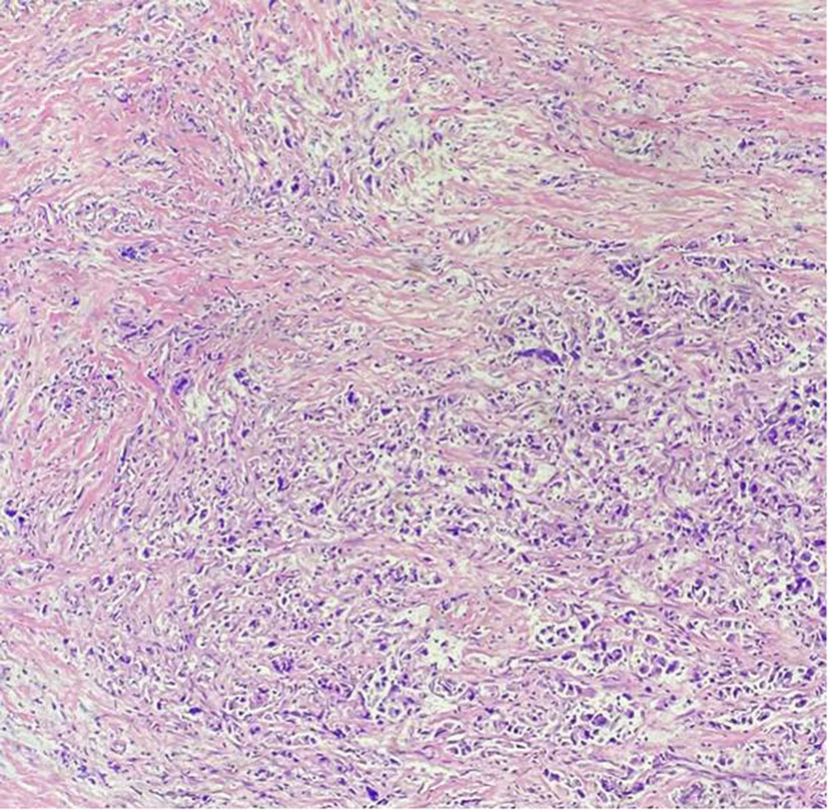
Pathology results. H&E staining of the biopsy specimen showed poorly differentiated adenocarcinoma.

**Figure 2:**
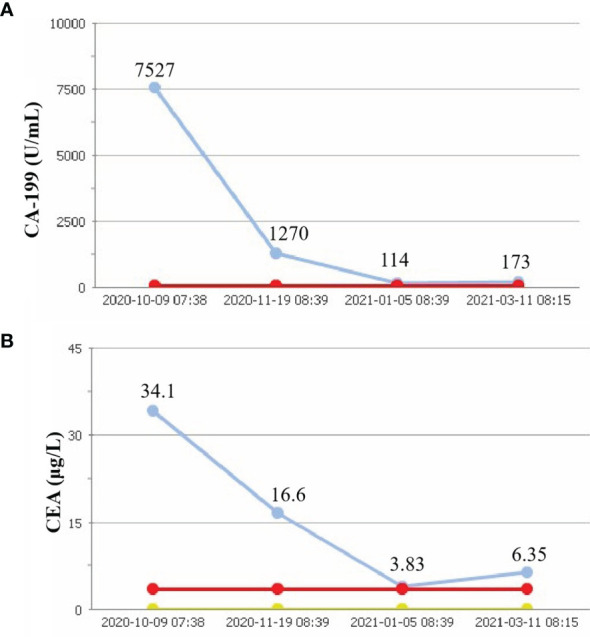
Tumor parameters. CA-199 concent **(A)**. CEA consent **(B)**. Red line, upper limits of normal; yellow line, lower limits of normal; blue line, actual value.

**Figure 3:**
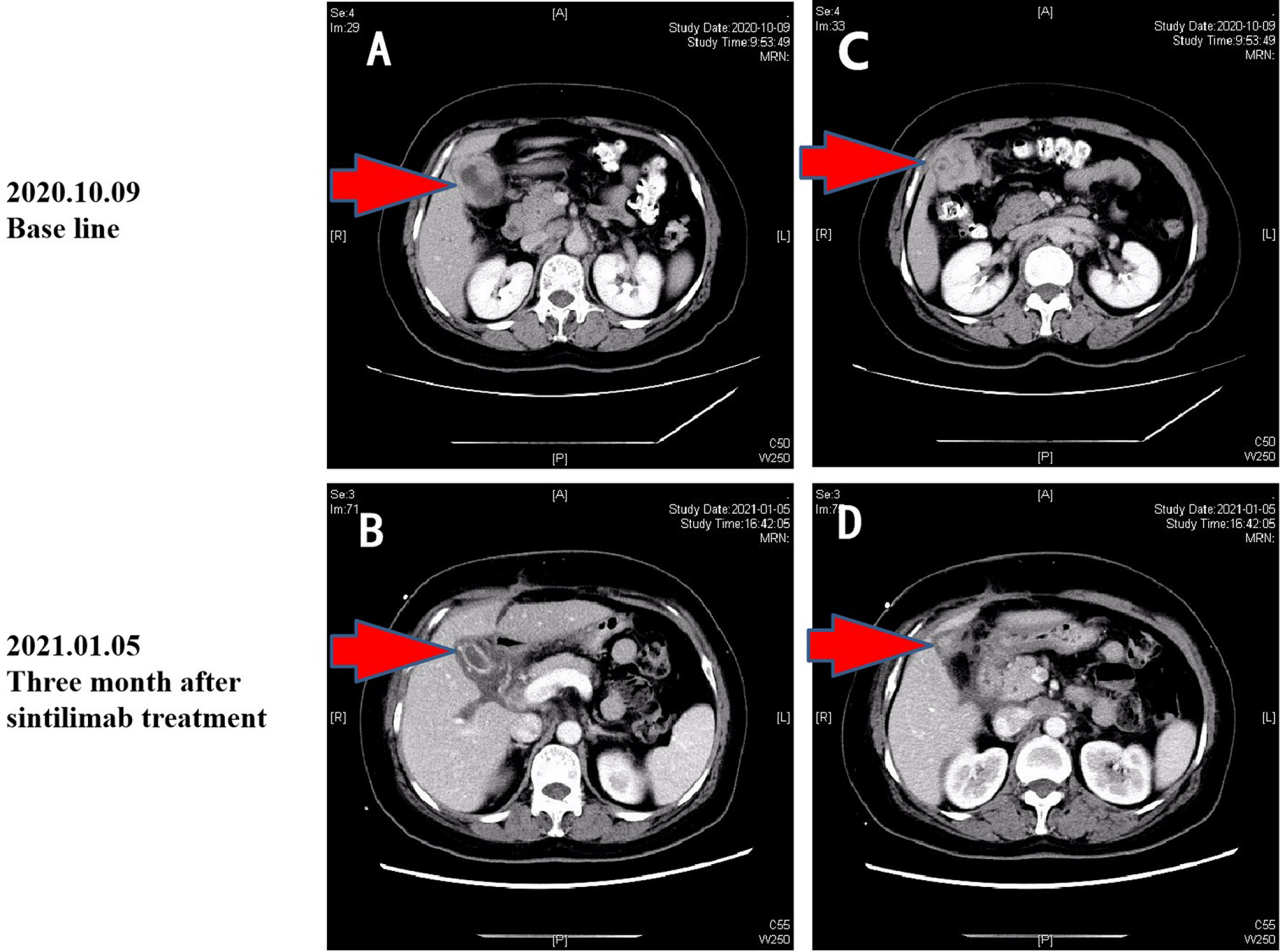
CT images of baseline **(A**, **C)** and after sintilimab treatment **(B**, **D)**.

**Figure 4:**
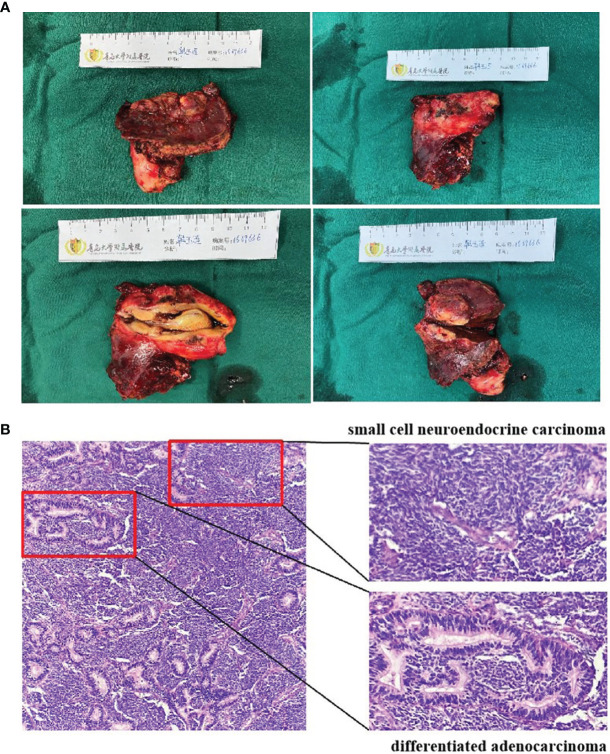
Surgical photo **(A)**. Pathology results **(B)**. H&E staining of the surgical specimen showed mixed neuroendocrine–nonneuroendocrine tumors.

On October 7, 2020, a 56-year-old woman admitted to our hospital due to right upper abdominal pain of 3 days duration. Abdominal computed tomography (CT) revealed space-occupying lesions at the bottom of the gallbladder, and space-occupying lesions in the hilar bile duct with intrahepatic bile duct dilatation, indicating malignant lesions. Multiple enlarged lymph nodes were mainly detected in the hepatic hilar and retroperitoneum. The levels of tumor markers were as follows: carcinoembryonic antigen (CEA), 34.1 μg/L, and carbohydrate antigen CA199, 7527 U/ml. Due to jaundice, the patient planned to undergo cholecystojejunostomy and R1 resection. The patient underwent exploratory laparotomy under general anesthesia on October 12, 2020. However, the patient had extensive lymph node metastasis and was not suitable for operation, then the patient underwent percutaneous transhepatic cholangial drainage (PTCD). The postoperative pathology showed that the tumor adjacent to the gallbladder was poorly differentiated adenocarcinoma with intrahepatic metastasis, classified as clinical stage T3N2M0 according to the tumor-node-metastasis (TNM) staging of the American Joint Committee on Cancer (AJCC) (**Figure 1**). Next-generation sequencing (NGS) revealed that these mutations were STK11, RB1, CTNNA3, KEAP1, RBM10, and TCF7L2; moreover, PD-L1 positivity was observed. The patient refused chemotherapy, therefore, considering the high positive score, Combined Proportion Score (CPS 98) and Tumor Proportion Score (TPS 90%), sintilimab was given 200 mg every 3 weeks for eight cycles from November 2, 2020, and no adverse reactions were observed. Two weeks after the first administration, the level of tumor markers decreased: CEA, 16.6 μg/L, and CA199, 1,270 U/ml (**Figure 2**). The patient reexamined after three cycles of treatment. CT showed that the size of tumor and lymph node significantly reduced (**Figure 3**). Moreover, the patient’s jaundice subsided and PTCD was removed. Meanwhile, CEA and CA199 decreased significantly to 3.83 μg/L and 114 U/ml, respectively (**Figure 2**). However, on March 11, 2021, CT indicated disease progression after six cycles of sintilimab treatment, accompanied by elevation of CA199 to 173 U/ml and CEA to 6.35 μg/L. The combination therapy consisting of sintilimab and Tegafur (chemotherapeutic drug) administered 3 days later. After eight treatment cycles, the patient’s CA199 level increased to 222 U/ml on April 13, 2021. One week later, CT revealed local liver metastasis, and the levels of CEA and CA 199 were 14.5 μg/L and 333 U/ml, respectively. Considering the progress of the disease, the entire liver metastasis and gallbladder were removed, and the lymph node dissection in the first porta hepatis was performed (**Figure 4A**). Postoperative pathology showed a poorly differentiated malignant tumor of the gallbladder, consistent with mixed neuroendocrine–nonneuroendocrine tumors (poorly differentiated adenocarcinoma accounted for 60%, small cell neuroendocrine carcinoma accounted for about 40%) (**Figure 4B**). Considering the difference from the first pathological diagnosis, the second NGS (liver metastasis, small cell neuroendocrine carcinoma) revealed that the mutations were PIK3CA, CDKN2A, CDKN2B, TP53, RB1, CCNE1, CEBPA, CTNNA3, GRM3, KEAP1, PBX1, PRKDC, RASA1, RBM10, and TBX3. Furthermore, PD-L1 positivity was also observed (CPS 10; TPS 8%). Different pathological diagnosis showed different mutation profiles. At present, the patient has been in a stable state with better life quality and is still in continuous sintilimab and chemotherapy.

## Discussion

In this case, the patient was diagnosed with gallbladder adenocarcinoma for the first time; NGS carried out at the beginning shows the mutation of STK11 (S216F), the loss-of-function mutations. A retrospective analysis found that patients with nonsmall-cell lung cancer with STK11 had poor efficacy in the treatment with PD-1/PD-L1 inhibitors, and the progression-free survival period was shortened compared with patients without mutation (4). However, this evidence was not found in gallbladder adenocarcinoma. Meanwhile, the patient had high PD-L1 expression (CPS 98; TPS 90%). The research in 2020 showed that PD-L1 overexpression (tumor proportion score ≥50%) in patients with refractory advanced biliary tract cancer was significantly associated with higher response rates (*vs*. <50%; 37.5% *vs*. 6.5%; *p* = 0.049) (5). This suggests that PD-L1 may be a prognostic biomarker, and anti-PD-1/PD-L1 immunotherapy may promise a PD-L1-positive advanced solid tumor. Previous studies have shown that pembrolizumab monotherapy provides durable antitumor activity in 13% of patients with 24 BTCs (including four GBCs) (6). A multi-institutional study found that nivolumab was well tolerated and showed modest efficacy with durable response in patients with refractory BTC (7). The above studies have indicated that immunotherapy had potential clinical application in the treatment of BTCs. Sintilimab is a humanized and highly selective monoclonal antibody, which can block the interaction between PD-1 and its ligand. The drug has been approved in China to treat relapsed or refractory classical Hodgkin’s lymphoma and been tested on several advanced solid tumors. Therefore, the patient with high PD-L1 expression achieved partial remission after three courses of sintilimab monotherapy, and CT showed a perfect clinical effect. 

Many studies have shown that ICIs have a synergistic effect with chemotherapy in solid tumors. However, there have been few reports of this combination therapy in advanced BTCs. In a small sample study, 32 patients with unresectable or metastatic biliary tract cancer (including six patients with gallbladder cancer) were recruited. In treatment with nivolumab plus gemcitabine and cisplatin, 15 (55.6%) patients achieved an objective response, including five (18.6%) with a complete response (CR), and the DCR was 92.6% (8). Similarly, camrelizumab combined with chemotherapy as first-line treatment for metastatic BTCs showed acceptable safety and efficacy (9). Based on the above clinical studies, the patient joined the Tegafur treatment strategy after the progress of immune monotherapy, and finally achieved an excellent therapeutic effect.

Gallbladder MiNENs present with similar symptoms and are in the same age group as do carcinomas. Specific management and treatment guidelines have not been established since MiNENs are very rare. It is highly invasive regardless of the size of the tumor (10). Such lesions can occur in any organ originating from the primitive intestine. The origin of this type of malignant tumor is still unclear. Like other gallbladder tumors, MiNENs has no specific symptoms, which is the main reason why they are always diagnosed in the late stage of the disease. The combination of immunotherapy and chemotherapy is effective in this case, which provides a feasible and novel treatment option for MiNENs of gallbladder patients with the positive expression of PD-L1 in the future.

## Data Availability Statement

The original contributions presented in the study are included in the article/supplementary material. Further inquiries can be directed to the corresponding author.

## Ethics Statement

The studies involving human participants were reviewed and approved by the Medical Ethics Committee of The Affiliated Hospital of Qingdao University. The patients/participants provided their written informed consent to participate in this study. Written informed consent was obtained from the individual(s) for the publication of any potentially identifiable images or data included in this article.

## Author Contributions

XH, ZY, YG, and YZ collected and organized the data. CL wrote the manuscript. LW, JC, and LL prepared the figures. NF critically revised the manuscript for intellectual content. All authors contributed to the article and approved the submitted version.

## Funding

This study was funded by the Clinical Medicine + X Project of Qingdao University (Grant number QDFY+X2021023).

## Conflict of Interest

Authors LL and YZ were employed by Technology Co. Ltd.

The remaining authors declare that the research was conducted in the absence of any commercial or financial relationships that could be construed as a potential conflict of interest.

## Publisher’s Note

All claims expressed in this article are solely those of the authors and do not necessarily represent those of their affiliated organizations, or those of the publisher, the editors and the reviewers. Any product that may be evaluated in this article, or claim that may be made by its manufacturer, is not guaranteed or endorsed by the publisher.
